# Rituximab as Salvage Therapy in Difficult-to-Treat Autoimmune Hepatitis: A Prospective Case Series

**DOI:** 10.1016/j.gastha.2026.101095

**Published:** 2026-08-18

**Authors:** Craig Lammert, Nadia Blessing, Maaz Arif, Divya Minnaganti, Regina Weber, Kelsey Green, Raj Vuppalanchi, Naga Chalasani

**Affiliations:** Division of Gastroenterology and Hepatology, Department of Medicine, Indiana University School of Medicine, Indianapolis, Indiana

**Keywords:** Autoimmune Hepatitis, Rituximab, Refractory Liver Disease, B-Cell-Directed Therapy, Corticosteroid-Sparing

## Abstract

**Background and Aims:**

A subset of patients with autoimmune hepatitis (AIH) fail to achieve or sustain a complete biochemical response (CBR) to standard immunosuppression, resulting in a significant unmet clinical need. Rituximab, an anti-CD20 monoclonal antibody, has been investigated in refractory AIH, but prospective data remain limited. We sought to characterize the biochemical response and corticosteroid-sparing effects of rituximab in patients with difficult-to-manage AIH.

**Methods:**

We report a prospective case series of adults with difficult-to-manage AIH enrolled in the Genetic Repository of Autoimmune Liver Disease and Contributing Exposures cohort at Indiana University and treated with rituximab (1000 mg intravenously on days 1 and 15). Eligible patients had refractory disease, corticosteroid dependence, or inadequate biochemical control. Response was assessed at 12, 24, and 48 weeks. CBR was defined as normalization of alanine aminotransferase, aspartate aminotransferase, and immunoglobulin G. Analyses of biochemical outcomes were restricted to patients with active baseline disease.

**Results:**

Seven patients were enrolled; 6 had abnormal baseline biochemical indices. Biochemical improvement at 24 weeks was observed in all 6 patients with active baseline disease (100%), with 1 patient (16.7%) achieving CBR. However, response durability was limited, and biochemical worsening between 24 and 48 weeks occurred in 4 of 6 patients (66.7%). Response patterns included insufficient response with subsequent attenuation (n = 4), sustained CBR (n = 1), and early response followed by relapse (n = 1). Median prednisone-equivalent dose decreased from 30 mg/day at baseline to 12.5 mg/day at 48 weeks (−58.3%). No serious adverse events, infections, or hepatic decompensation occurred.

**Conclusion:**

In this prospective cohort of difficult-to-manage AIH, rituximab produced early biochemical improvement and a sustained corticosteroid-sparing effect, but a durable biochemical remission was uncommon. These data support further prospective evaluation of rituximab and next-generation B-cell-directed therapies in refractory AIH.

## Introduction

Autoimmune hepatitis (AIH) is a chronic immune-mediated liver disease characterized by progressive hepatocellular inflammation, circulating autoantibodies, and elevated immunoglobulin G (IgG) levels.^1^ If untreated, AIH can lead to cirrhosis, liver failure, and death. Standard first-line therapy consists of corticosteroids, often in combination with azathioprine, and is effective in most patients.^1^^,^^2^ However, a substantial subset of individuals develops treatment intolerance, corticosteroid dependence, or inadequate biochemical response, necessitating alternative therapeutic strategies.^1^^,^^2^

Second-line and third-line immunosuppressive agents, including mycophenolate mofetil, calcineurin inhibitors, and other adjunctive therapies, are commonly used in patients with difficult-to-manage disease. Although these therapies can be effective in selected cases, many patients fail to achieve complete biochemical remission or experience ongoing disease activity despite multi-agent immunosuppression. Importantly, failure to achieve a complete biochemical response has been associated with worse long-term outcomes, including progression to cirrhosis and liver-related mortality. These challenges highlight an unmet need for targeted therapies in refractory AIH.^3^, ^4^, ^5^

B cells have emerged as key contributors to the pathogenesis of AIH through roles in autoantibody production, antigen presentation, and modulation of T-cell responses.^6^ Rituximab, a chimeric monoclonal antibody targeting CD20-positive B cells, has been increasingly explored as a therapeutic option in refractory autoimmune diseases, including AIH.^6^^,^^7^ Early case reports and small series have demonstrated biochemical improvement following rituximab therapy in patients with treatment-resistant disease.^8^, ^9^, ^10^, ^11^ Subsequent retrospective cohort studies and registry-based analyses have supported its possible efficacy and safety; however, response durability and patterns of relapse remain incompletely defined.^12^^,^^13^ In particular, existing studies have largely focused on aggregate biochemical outcomes, with limited characterization of individual patient trajectories and temporal response patterns following B-cell depletion. Given the heterogeneity of AIH and variability in the treatment response, a more granular understanding of clinical response phenotypes is needed to inform patient selection and refine therapeutic strategies.

The B-cell compartment in AIH is heterogeneous and extends well beyond autoantibody secretion. Autoreactive B cells in AIH amplify hepatic inflammation by presenting antigens to CD4+ T cells, secreting pro-inflammatory cytokines, including interleukin-6 and tumor necrosis factor-alpha, and impairing regulatory T-cell function.^6^ A critical distinction in B-cell biology is the differential expression of surface markers across B-cell maturation stages: CD20 is expressed from the late pro-B cell stage through memory B cells but is downregulated during terminal differentiation into plasmablasts and long-lived plasma cells. This expression pattern has important therapeutic implications, as CD20-directed depletion with rituximab spares the most terminally differentiated antibody-secreting cells, which may sustain pathogenic IgG production and contribute to incomplete or non-durable responses.^6^ By contrast, CD19 is expressed across a broader continuum of B-cell differentiation, including plasmablasts, making it a potentially more comprehensive therapeutic target. B-cell survival is further maintained by the cytokines B-cell activating factor (BAFF) and a proliferation-inducing ligand (APRIL), both of which are elevated in AIH and promote B-cell maturation, autoantibody production, and resistance to apoptosis.^6^^,^^14^ These survival signals remain active even in the setting of CD20 depletion, providing a mechanistic basis for the historically incomplete and transient responses observed with rituximab.^6^^,^^15^

In this context, we conducted an investigator-initiated, open-label prospective case series of rituximab therapy in patients with difficult-to-manage AIH. We aimed to characterize not only the biochemical response but also the temporal dynamics and durability of the treatment effect, with a particular focus on clinical trajectories, individual response phenotypes, and durability of the effect over 48 weeks of follow-up.

## Methods

### Study Design and Patient Population

We conducted an open-label, prospective case series of rituximab therapy in adult patients with difficult-to-manage AIH enrolled in the Genetic Repository of Autoimmune Liver Disease and Contributing Exposures cohort at Indiana University. This therapy was provided as part of escalating clinical care. Patients were followed for 48 weeks after treatment using predefined clinical, biochemical, and safety assessments. Eligible patients had biopsy-proven AIH and were included if they had inadequately controlled disease despite standard immunosuppressive therapy or were corticosteroid-dependent. Refractory disease was defined as persistent elevation of aminotransferases despite treatment with corticosteroids and at least one additional immunosuppressive agent, without medication changes, for 3 months prior to enrollment. Corticosteroid dependence was defined as the inability to taper corticosteroids without biochemical relapse.^1^^,^^16^ Physical examinations were performed as part of routine clinical care at each visit. Findings were not systematically recorded as study outcomes, and no clinically significant changes on physical examination were identified during follow-up.

### Ethical Issues and Consent

This study was conducted in accordance with the ethical principles of the Declaration of Helsinki and was approved by the institutional review board of Indiana University (Protocol #1903320072). All patients provided written informed consent prior to participation. Given that rituximab was administered as part of standard escalating clinical care, no additional regulatory approvals beyond institutional review board oversight were required. Patients were counseled about the risks and possible benefits of rituximab therapy, including infectious risks and uncertainties regarding treatment durability.

### Treatment Protocol

All patients received rituximab administered intravenously at a dose of 1000 mg on day 1 and day 15. Concomitant immunosuppressive therapies, including corticosteroids and other agents, were continued and adjusted at the treating physician's discretion based on clinical response. To standardize corticosteroid exposure across patients, all corticosteroid doses were converted to prednisone equivalents. Budesonide was converted using an assumed equivalence of 9 mg budesonide to 30 mg prednisone.

### Data Collection

Clinical and laboratory data were collected at baseline and at 12, 24, and 48 weeks following administration of rituximab. Data included demographic characteristics, disease duration, prior and concomitant therapies, and laboratory parameters including alanine aminotransferase (ALT), aspartate aminotransferase (AST), IgG, alkaline phosphatase, bilirubin, albumin, and creatinine. Liver fibrosis was assessed using vibration-controlled transient elastography, with cirrhosis defined as a liver stiffness measurement (LSM) 15 kPa.

### Outcomes and Definitions

The primary outcome was biochemical response at 12, 24, and 48 weeks following rituximab therapy. Complete biochemical response was defined as normalization of ALT (<45 U/L), AST (<45 U/L), and IgG (<1500 mg/dL). Patients who did not meet the criteria for a complete biochemical response were classified as having an insufficient or no response. Insufficient biochemical response was defined as improvement in ALT, AST, and/or IgG without normalization, while no response was defined as no improvement or worsening of biochemical parameters. Biochemical worsening between 24 and 48 weeks was defined as an increase in ALT, AST, or IgG relative to values at week 24.^17^

### Statistical Analysis

Given the small sample size, analyses were descriptive. Continuous variables are reported as median values with quartile 1 (Q1) and quartile 3 (Q3). Categorical variables are presented as counts and percentages. Analyses of biochemical response were restricted to patients with abnormal baseline biochemical indices (ALT and/or AST >45 U/L and/or elevated IgG >1500 mg/dL), representing the at-risk population for treatment response.

## Results

### Patient Characteristics

**Figure 1:**
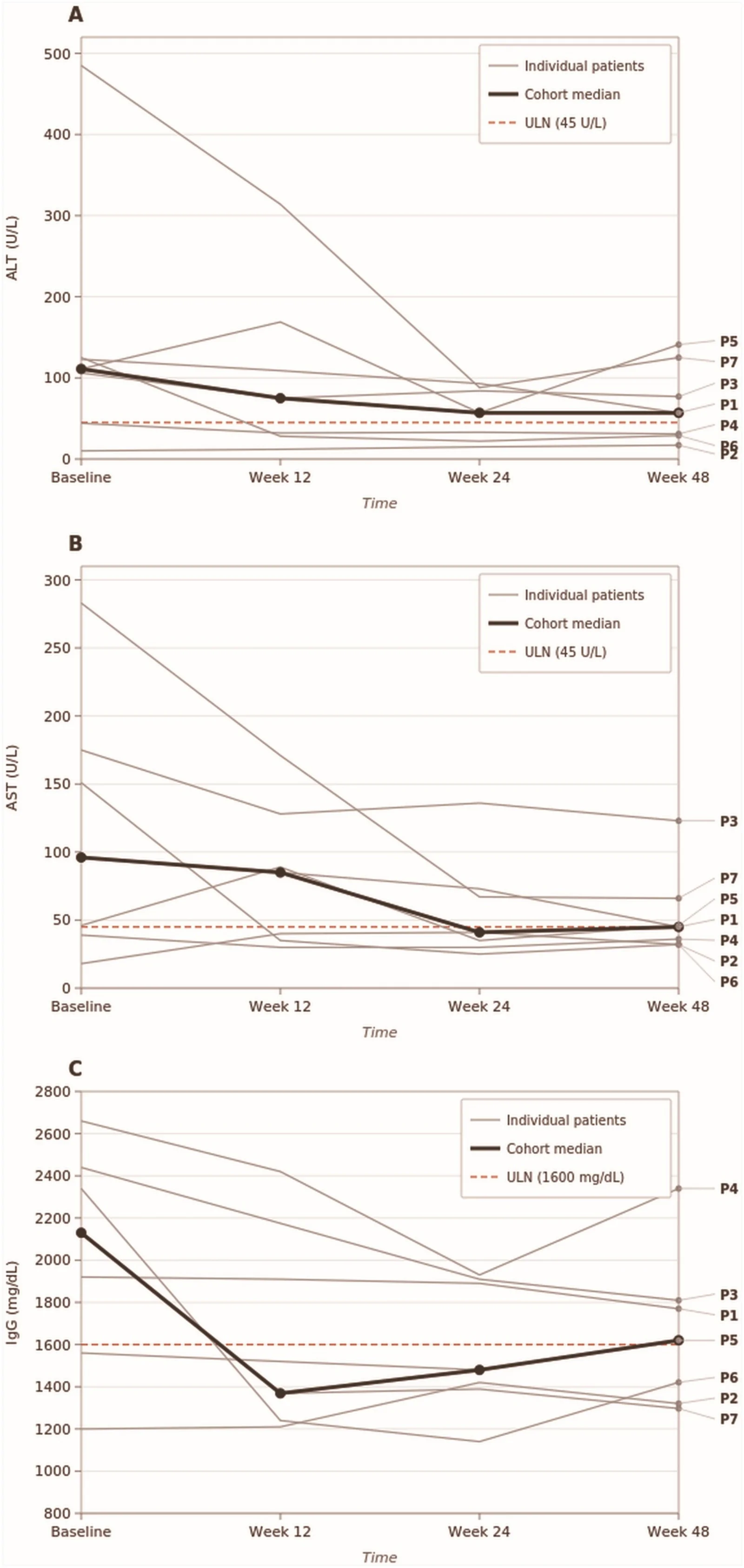
(A–C) Longitudinal changes in liver biochemistries and IgG following rituximab therapy in patients with difficult-to-manage autoimmune hepatitis. Individual patient trajectories of (A) alanine aminotransferase (ALT), (B) aspartate aminotransferase (AST), and (C) immunoglobulin G (IgG) levels measured at baseline, 12 weeks, 24 weeks, and 48 weeks following rituximab administration. Each gray line represents an individual patient (n = 7), with patient identifiers (P1–P7) labeled at both the start and end of each trajectory. The bold black line and filled circles represent the cohort median at each time point. Overall, reductions in ALT, AST, and IgG were observed in the majority of patients by 12 and 24 weeks, with the greatest group-level improvement observed at 24 weeks. A subset of patients demonstrated biochemical worsening by 48 weeks. ULN, 45 U/L

Seven patients with difficult-to-manage AIH were treated with rituximab and completed follow-up through 48 weeks after the second infusion. Baseline demographic, clinical, and serologic characteristics are summarized in Table 1. Abnormal liver tests (ALT and/or AST >45 U/L) were present in 6 of 7 patients (85.7%) at baseline, and elevated serum IgG levels were observed in 5 of 7 patients (71.4%) (Figure 1A–C).

**Table 1:** Individual Patient Clinical Characteristics at Baseline and Follow-Up

Patient	Sex	Race	Age (y) at rituximab start	Age at AIH diagnosis (y)	Disease duration before rituximab (y)	Cirrhosis at baseline	MELD at baseline	Total bilirubin (mg/dL)	Albumin (g/dL)	ANA titer	ASMA titer	Immunosuppression at baseline	Immunosuppression at 12 mo	Prior immunosuppressive agents	Corticosteroid at baseline	Corticosteroid at 6 mo	Corticosteroid at 12 mo
P1	Female	White	28	15	13	Yes	8	0.8	3.9	Negative	1:40	Mycophenolic acid, cyclosporine	Sirolimus, cyclosporine	Azathioprine, mycophenolic acid	Prednisone 35 mg	Prednisone 35 mg	Prednisone 50 mg
P2	Female	White	54	52	2	No	8	0.4	3.6	1:40	1:160	Azathioprine, tacrolimus	Tacrolimus	Azathioprine	Prednisone 40 mg	Prednisone 10 mg	Prednisone 25 mg
P3	Male	Whie	35	18	7	Yes	9	1.8	2.9	1:320	—	Azathioprine, sirolimus	Azathioprine, sirolimus	—	Prednisone 10 mg	Prednisone 10 mg	Prednisone 5 mg
P4	Female	Black	47	37	10	Yes	9	0.7	3.7	1:5120	1:320	Mycophenolate mofetil	Mycophenolate mofetil	Azathioprine, tacrolimus	Prednisone 20 mg	Prednisone 15 mg	Prednisone 12.5 mg
P5	Male	White	25	21	4	No	7	0.5	4.3	1:640	1:1280	Mycophenolate mofetil, tacrolimus	Mycophenolate mofetil, tacrolimus	—	Prednisone 30 mg	Prednisone 30 mg	Prednisone 30 mg
P6	Male	White	54	53	1	Yes	15	1.5	3.4	1:160	1:640	Mycophenolate mofetil	Mycophenolate mofetil	—	Prednisone 40 mg	Prednisone 15 mg	Prednisone 10 mg
P7	Female	White	21	19	2	Yes	6	0.9	3.8	1:640	Negative	Mycophenolate mofetil, sirolimus	Mycophenolate mofetil, sirolimus	Azathioprine, tacrolimus	Prednisone 10 mg	Prednisone 10 mg	Prednisone 10 mg

Cirrhosis is defined as a liver stiffness measurement (LSM) ≥15 kPa on vibration-controlled transient elastography. All corticosteroid doses are reported as prednisone-equivalent daily doses. ANA and ASMA titers reflect values obtained at or near the time of rituximab initiation. "—" denotes no prior use or not applicable.

All patients were non-Hispanic.

ANA, antinuclear antibody; ASMA, anti-smooth muscle antibody; MELD, Model for End-Stage Liver Disease.

At study entry, the median ALT was 111 U/L (quartile 1 [Q1]: 44 U/L; quartile 3 [Q3]: 125 U/L), AST 96 U/L (Q1: 39 U/L; Q3: 175 U/L), and alkaline phosphatase 91 U/L (Q1: 66 U/L; Q3: 141 U/L). Median IgG was 1920 mg/dL (Q1: 1370 mg/dL; Q3: 2440 mg/dL). Synthetic liver function was preserved across the cohort, with a median total bilirubin of 0.8 mg/dL (Q1: 0.5 mg/dL; Q3: 1.5 mg/dL) and albumin of 3.7 g/dL (Q1: 3.4 g/dL; Q3: 3.9 g/dL).

Noninvasive fibrosis assessment demonstrated advanced disease in a substantial proportion, with a median LSM of 21.6 kPa (Q1: 13.4 kPa; Q3: 25.7 kPa), and 5 of 7 patients (71.4%) meeting criteria for cirrhosis (LSM ≥15 kPa). The median controlled attenuation parameter score was 248 dB/m (Q1: 188 dB/m; Q3: 301 dB/m).

The median duration of AIH prior to rituximab therapy was 4 years (Q1: 2 years; Q3: 11 years). All patients were receiving corticosteroids at baseline (median prednisone-equivalent dose, 30 mg/day [Q1: 10 mg/day; Q3: 40 mg/day]) and were on concomitant immunosuppressive therapy. The primary indication for rituximab was refractory or inadequately controlled disease in 5 of 7 patients (71.4%) and corticosteroid dependence in 2 of 7 patients (28.6%), reflecting a highly treatment-resistant population.

### Clinical Course Following Rituximab

Clinical responses to rituximab were heterogeneous, with most patients showing biochemical improvement by 24 weeks and attenuation of response by 48 weeks (Figure 1A–C) (Table 2). Among patients with active biochemical disease at baseline, 6 (100%) demonstrated improvement in AST, ALT, and/or IgG by 24 weeks; however, 4 of 6 (66.7%) exhibited biochemical worsening by 48 weeks. Notably, one patient (16.7%) maintained complete biochemical remission at 48 weeks, despite partial elevation in biochemistries above nadir values.

**Table 2:** Individual Patient Response Trajectories Following Rituximab Therapy

Patient	Indication for rituximab	Biochemical response at 24 wk	Biochemical response at 48 wk	Response pattern
P1	Refractory	Insufficient response	Insufficient response	Insufficient biochemical response with steroid escalation
P2	Corticosteroid-dependent	Stable response	Stable response	Stable disease and steroid reduction
P3	Refractory	Insufficient response	Insufficient response	Insufficient biochemical response and steroid reduction
P4	Corticosteroid-dependent	Insufficient response	Insufficient response	Insufficient biochemical response and steroid reduction
P5	Refractory	No response	No response	No response and steroid dose stable
P6	Refractory	Complete remission	Complete remission	Complete biochemical response with steroid reduction
P7	Refractory	Insufficient response	Insufficient response	Insufficient response and steroid dose stable

### Individual Patient Trajectories

Patient 1 was a 28-year-old woman with a 13-year history of AIH and established cirrhosis (LSM 17.2 kPa) who had failed azathioprine and remained on multi-agent immunosuppression with persistent aminotransferase elevation (ALT 123 U/L, AST 96 U/L). She demonstrated early biochemical improvement by 24 weeks but experienced attenuation of response by 48 weeks, requiring corticosteroid dose escalation to prednisone 50 mg/day.

Patient 2 was a 54-year-old woman with a 2-year history of non-cirrhotic AIH (LSM 13.4 kPa) treated for corticosteroid dependence, with near-normal aminotransferases at baseline (ALT 10 U/L, AST 18 U/L) and prednisone 40 mg/day despite azathioprine and tacrolimus. She remained biochemically stable through 48 weeks without hepatic deterioration, though corticosteroid discontinuation was not achieved.

Patient 3 was a 35-year-old man with advanced cirrhosis (LSM 38.5 kPa) and persistently elevated aminotransferases (ALT 106 U/L, AST 175 U/L) and IgG (2440 mg/dL) despite azathioprine and sirolimus. Early biochemical improvement was observed by 24 weeks with successful corticosteroid reduction (prednisone 10 → 5 mg/day); by 48 weeks, partial worsening had occurred, but aminotransferases remained below baseline.

Patient 4 was a 47-year-old woman with cirrhosis (LSM 25.7 kPa), corticosteroid dependence, and markedly elevated IgG (2660 mg/dL) with high-titer autoantibodies (antinuclear antibody 1:5120, anti-smooth muscle antibody 1:320), despite near-normal aminotransferases. She demonstrated partial reductions in IgG and aminotransferases without normalization throughout follow-up, with modest corticosteroid reduction (prednisone 20 → 12.5 mg/day at 12 months).

Patient 5 was a 25-year-old man with non-cirrhotic AIH (LSM 6.8 kPa) and active disease (ALT 111 U/L) on mycophenolate mofetil, tacrolimus, and prednisone 30 mg/day. He demonstrated minimal biochemical changes at any time point, with ALT and IgG essentially unchanged at 48 weeks and no reduction in corticosteroid requirement; he remained on prednisone 30 mg/day throughout.

Patient 6 was a 54-year-old man with cirrhosis (LSM 21.6 kPa, Model for End-Stage Liver Disease 15) and markedly elevated aminotransferases (ALT 125 U/L, AST 151 U/L) on high-dose prednisone (40 mg/day) and mycophenolate mofetil. He exhibited a strong early biochemical response with a successful steroid tapering to prednisone 15 mg/day by 24 weeks. He reached complete biochemical remission at 24 weeks. At 48 weeks, partial worsening was observed, with mild elevations in ALT, AST, and IgG above nadir values; however, all parameters remained below his baseline levels.

Patient 7 was a 21-year-old woman with cirrhosis (LSM 22.4 kPa) and the highest baseline aminotransferases in the cohort (ALT 485 U/L, AST 283 U/L) on sirolimus, mycophenolate mofetil, and prednisone 10 mg/day. She demonstrated a strong early biochemical response by 24 weeks; at 48 weeks, a partial worsening had occurred, but aminotransferases remained well below baseline.

### Biochemical Response to Rituximab

Longitudinal changes in ALT, AST, and IgG are shown in Figure 1A–C and in Table 1. Group-level reductions in aminotransferases and IgG were most pronounced at 24 weeks, with improvement in ALT in 6 of 7 patients (85.7%) and in IgG in 5 of 7 patients (71.4%). By 48 weeks, ALT normalization (below the upper limit of normal, 45 U/L) was observed in only 3 of 7 patients (42.9%), and ALT remained below baseline in 5 of 7 patients (71.4%), reflecting the limited durability of complete biochemical normalization across the cohort.

Among the 6 patients with abnormal baseline biochemical indices, formal biochemical response categorization at 24 weeks identified 1 of 6 (16.7%) with a complete biochemical response (normalization of ALT, AST, and IgG), 4 of 6 (66.7%) with an insufficient biochemical response, and 1 of 6 (16.7%) with no response. By 48 weeks, 1 of 6 (16.7%) patients maintained a complete biochemical response, and 4 of 6 (66.7%) had an insufficient response, underscoring the limited durability of the treatment effect.

#### Corticosteroid reduction

**Figure 2:**
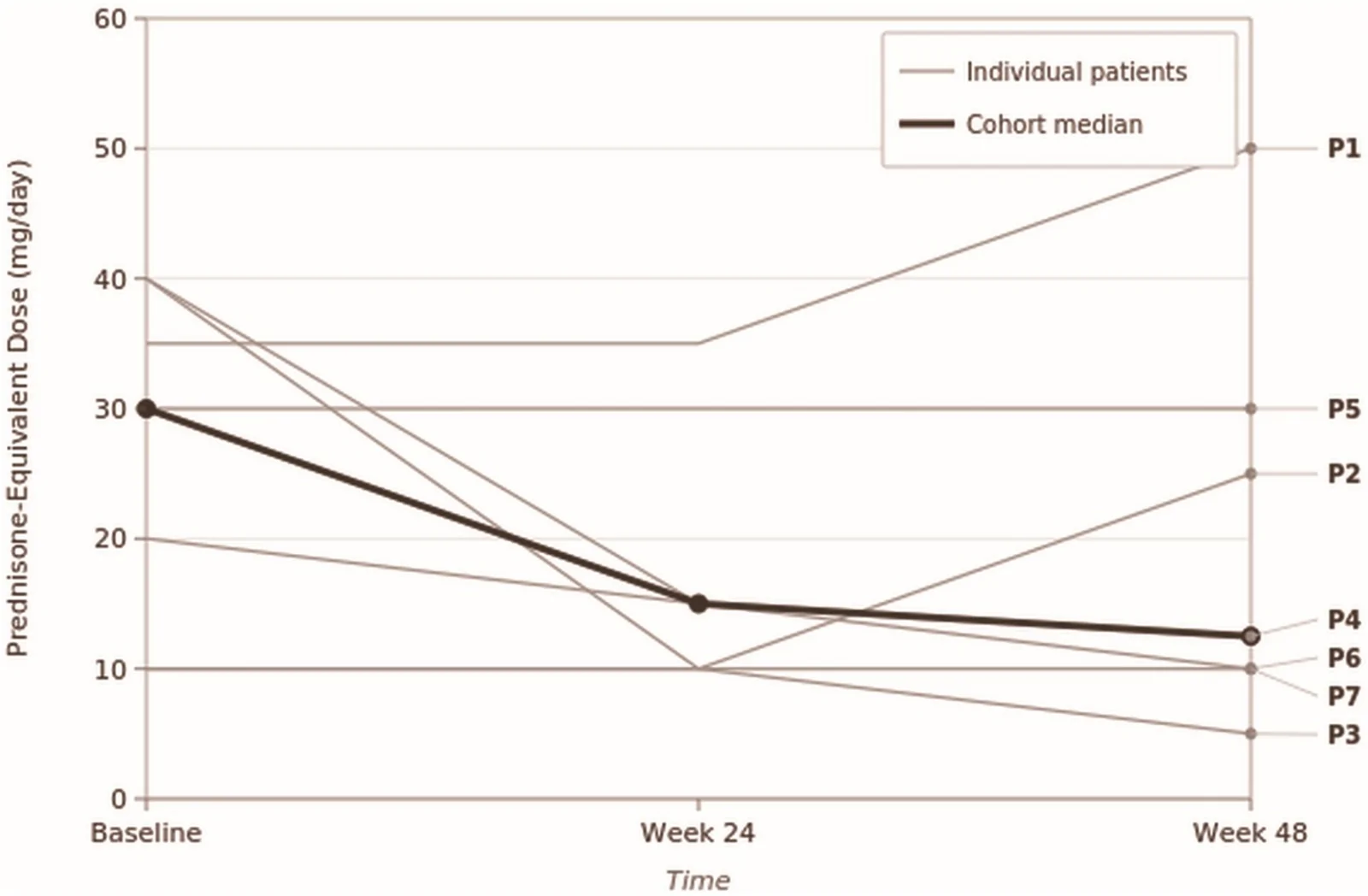
Longitudinal changes in prednisone-equivalent corticosteroid dose following rituximab therapy in patients with difficult-to-manage autoimmune hepatitis. Individual patient trajectories of prednisone-equivalent corticosteroid dose at baseline, 24 weeks, and 48 weeks following rituximab therapy. Each gray line represents an individual patient (n = 7), with patient identifiers (P1–P7) labeled at both the start and end of each trajectory. The bold black line and filled circles represent the cohort median at each time point. Median prednisone-equivalent dose decreased from 30 mg/day (Q1: 10, Q3: 40) at baseline to 15 mg/day (Q1: 10, Q3: 30) at 24 weeks, with a value of 12.5 mg/day (Q1: 10, Q3: 30) at 48 weeks.

At baseline, all patients received corticosteroid therapy with a median prednisone equivalent dose of 30 mg/day (Q1: 10 mg/day; Q3: 40 mg/day) (Figure 2). At 24 weeks, the median prednisone equivalent dose decreased to 15 mg/day (Q1: 10 mg/day; Q3: 30 mg/day), representing a 50% reduction from baseline. Corticosteroid dose reduction was observed in 3 out of 7 patients (42.9%). At 48 weeks, the median prednisone equivalent dose was 12.5 mg/day (Q1: 10 mg/day; Q3: 30 mg/day), corresponding to a 58.3% reduction from baseline. Sustained corticosteroid reduction was observed in 5 of 7 patients (71.4%), with no patients achieving complete discontinuation.

### Cirrhosis and Treatment Response

Five of 7 patients (71.4%) met criteria for cirrhosis at baseline, while 2 of 7 patients (28.6%) did not. Among patients with abnormal baseline biochemical indices (n = 6), 5 of 6 patients (83.3%) had cirrhosis. At 24 weeks, among patients with cirrhosis, 1 of 5 (20.0%) achieved a complete biochemical response, and 4 of 5 (80.0%) demonstrated an insufficient biochemical response. The one patient without cirrhosis who had abnormal baseline biochemical indices demonstrated no response at 24 or 48 weeks.

## Discussion

Rituximab therapy in this open-label, prospective case series of patients with difficult-to-manage AIH was associated with early biochemical improvement, followed by progressive attenuation of response over time. Most patients demonstrated reductions in aminotransferases and/or IgG by 24 weeks; however, the durability of response was limited, with biochemical worsening observed in 66.7% of patients with active disease between 24 and 48 weeks. Rituximab was well tolerated, with no serious adverse events or infections observed during follow-up.

An important finding of this study is the identification of distinct clinical response phenotypes following rituximab therapy. While a subset of patients exhibited early biochemical improvement, this was often followed by loss of disease control, suggesting a time-limited therapeutic effect. Other patients showed partial biochemical responses lacking normalization, and one patient exhibited a clear relapse following an initial strong response. These patterns highlight the heterogeneity of the treatment response in AIH and suggest that rituximab may induce transient immunologic modulation rather than sustained disease remission in many patients.

These data are broadly consistent with previous reports of rituximab use in AIH. Early case reports and small case series showed biochemical improvement in patients with refractory disease, although long-term outcomes were variable. In a prospective pilot study, Burak et al^18^ reported biochemical improvement in most patients following rituximab with sustained responses observed in a subset. Similarly, the International Autoimmune Hepatitis Group retrospective cohort demonstrated reductions in aminotransferases and corticosteroid requirements following rituximab therapy, although relapse or incomplete response remained common. More recently, registry-based analyses, including the ColHai cohort, have confirmed that rituximab can be effective in selected patients but may not result in a durable remission across all individuals. These data, taken together, support the role of rituximab as a therapeutic option in refractory AIH while showing the variability in response durability.

The limited durability of response observed in this study is biologically plausible and likely reflects the mechanism of action of rituximab. As an anti-CD20 monoclonal antibody, rituximab induces B-cell depletion, but B-cell repopulation occurs generally within 6 to 12 months following treatment.^19^ This temporal pattern closely mirrors the clinical trajectory observed in this cohort, with improvement at 24 weeks followed by biochemical worsening by 48 weeks. These data suggest that sustained disease control may require repeated dosing or maintenance strategies, as has been demonstrated in other autoimmune conditions and in IgG4-related diseases.^20^^,^^21^

In addition to the biochemical outcomes, rituximab demonstrated a corticosteroid-sparing effect in this cohort. Median prednisone equivalent dose decreased by 50% at 24 weeks (30–15 mg/day) and remained reduced at 48 weeks (12.5 mg/day). No patient achieved total corticosteroid discontinuation. Despite incomplete discontinuation of corticosteroids, this reduction is clinically meaningful in a population characterized by corticosteroid dependence and treatment resistance. These data are consistent with previous reports indicating lowered corticosteroid exposure following rituximab therapy.^12^^,^^13^

Notably, response patterns appeared to differ by baseline disease characteristics. Patients with cirrhosis demonstrated lower rates of complete biochemical response and more frequent incomplete responses relative to non-cirrhotic patients, although partial biochemical improvement was seen across both groups. Furthermore, all patients with elevated baseline IgG had underlying cirrhosis, supporting the idea that persistent IgG elevation may reflect more advanced disease biology and be less responsive to immunomodulatory therapy. These findings underscore the importance of disease stage in predicting treatment response and could inform patient selection for targeted B-cell therapies.

The short-lived nature of biochemical improvement in this cohort raises fundamental questions about whether CD20-directed B cell depletion is a mechanistically sufficient strategy for sustained disease control in AIH. Rituximab depletes circulating and tissue-resident B cells expressing CD20, but long-lived plasma cells, which do not express CD20, can persist in bone marrow niches and continue to secrete pathogenic immunoglobulins. Moreover, B-cell repopulation following rituximab is characterized by an early return of transitional and naive B cells that may re-establish autoreactive clones, especially in the absence of concomitant tolerance induction. These limitations indicate that a more comprehensive or durable B-cell suppression may be required to achieve sustained remission in refractory AIH.

Several emerging B-cell-directed strategies may offer greater depth and durability of immunosuppression. Next generation anti-CD20 monoclonal antibodies, including obinutuzumab and ublituximab, produce deeper and more sustained B-cell depletion than rituximab in preclinical models and clinical autoimmune disease settings, though neither has been formally evaluated in AIH.^22^ Anti-CD19 agents represent a potentially broader approach, as CD19 is expressed across a wider range of B-cell maturation stages than CD20, including on plasmablasts. Obexelimab and inebilizumab, both anti-CD19 monoclonal antibodies currently in development for autoimmune diseases, have demonstrated efficacy in conditions such as neuromyelitis optica spectrum disorder and offer theoretical advantages by targeting antibody-secreting precursors that are spared by rituximab.^14^ Targeting the B-cell survival cytokines BAFF and APRIL represents an orthogonal strategy that may complement depletion-based approaches. BAFF is elevated in AIH and sustains both mature B cells and plasma cells through binding to receptors, including B-cell activating factor receptor, and transmembrane activator and calcium-modulator and cyclophilin ligand interactor. Belimumab, an approved anti-BAFF monoclonal antibody, has shown preliminary promise in small cohorts of patients with refractory AIH,^15^ and ianalumab, a humanized anti-B-cell activating factor receptor antibody, has been evaluated in a multicenter, randomized, placebo-controlled phase 2/3 trial in patients with AIH with incomplete response to standard therapy (NCT03217422).^23^ Dual BAFF/APRIL blockade with agents such as povetacicept may offer even broader suppression of the antibody-secreting compartment.^24^ Finally, CD19-targeted chimeric antigen receptor T-cell therapy has demonstrated the capacity to achieve deep and potentially durable B-cell eradication in refractory systemic autoimmune diseases, including systemic lupus erythematosus, systemic sclerosis, and antineutrophil cytoplasmic antibody-associated vasculitis, with emerging data suggesting this approach may induce immunological reset and drug-free remission.^25^^,^^26^ Whether such strategies can be safely applied in the context of AIH, particularly in patients with cirrhosis and baseline immune dysregulation, remains to be established.

This study has several strengths. It represents a prospective, investigator-initiated evaluation of rituximab in a well-characterized cohort of patients with difficult-to-manage AIH, with standardized follow-up and detailed biochemical assessment at predefined time points. The case series design allows for granular characterization of individual response trajectories, giving insight into treatment kinetics and durability that may not be captured in larger retrospective studies. Limitations should also be considered. The small sample size and single-center design limit generalizability. The absence of a control group precludes definitive assessment of treatment efficacy, and concomitant immunosuppressive therapies may confound interpretation of treatment response. Additionally, longer-term follow-up beyond 48 weeks is needed to better define durability and relapse patterns following rituximab therapy. Patient-reported outcomes were not collected in this study, which represents an additional limitation of the current case series.

## Conclusion

Rituximab was associated with early biochemical improvement and a beneficial safety profile in patients with difficult-to-manage AIH; however, the durability of response was limited, with most patients showing biochemical worsening by 48 weeks. These data support a role for B-cell-targeted therapy in refractory AIH while highlighting the need for optimized treatment strategies, including maintenance dosing approaches and refined patient selection, in future prospective studies.

## Supplementary Materials

CARE_checklist_completed
