# Supplementary material for: Rituximab as Salvage Therapy in Difficult-to-Treat Autoimmune Hepatitis: A Prospective Case Series
Source: Gastro Hep Adv. 2026 Aug 18;5(11):101095. doi: 10.1016/j.gastha.2026.101095 (PMC13584114; doi:10.1016/j.gastha.2026.101095)
Supplement: Supplementary file 1 [file mmc1.docx]

**CARE Checklist — Completed**

*Lammert et al. Rituximab as Salvage Therapy in Difficult-to-Treat Autoimmune Hepatitis: A Prospective Case Series*

Gastro Hep Advances — Submission

| **Topic** | **Item** | **Checklist Item Description** | **Reported on Line / Location** |
| --- | --- | --- | --- |
| **Title** | **1** | The diagnosis or intervention of primary focus followed by the words "case report" | Line 3 — "Rituximab as Salvage Therapy in Difficult-to-Treat Autoimmune Hepatitis: A Prospective Case Series" |
| **Key Words** | **2** | 2 to 5 key words that identify diagnoses or interventions in this case report, including "case report" | Line 25 — autoimmune hepatitis; rituximab; refractory liver disease; B-cell directed therapy; corticosteroid-sparing |
| **Abstract (no references)** | **3a** | Introduction: What is unique about this case and what does it add to the scientific literature? | Lines 55–57 — Background and Aims paragraph |
|  | **3b** | Main symptoms and/or important clinical findings | Lines 63–65 — Results paragraph (abstract) |
|  | **3c** | The main diagnoses, therapeutic interventions, and outcomes | Lines 63–65 — Results paragraph (abstract) |
|  | **3d** | Conclusion — What is the main "take-away" lesson(s) from this case? | Lines 67–69 — Conclusions paragraph (abstract) |
| **Introduction** | **4** | One or two paragraphs summarizing why this case is unique (may include references) | Lines 73–83 — Introduction (5 paragraphs covering AIH background, unmet need, B-cell biology, and study rationale) |
| **Patient Information** | **5a** | De-identified patient specific information | Lines 131–143 — Individual Patient Trajectories (P1–P7; age, sex, disease history provided; ethnicity not collected) |
|  | **5b** | Primary concerns and symptoms of the patient | Lines 131–143 — Chief complaint and disease activity for each patient described in individual vignettes |
|  | **5c** | Medical, family, and psycho-social history including relevant genetic information | Lines 131–143 — Medical history, comorbidities, and prior immunosuppressive therapy described per patient; family/psychosocial history not systematically collected |
|  | **5d** | Relevant past interventions with outcomes | Lines 131–143 and Table 1 (Lines 183–195) — Prior immunosuppressive agents and outcomes detailed per patient |
| **Clinical Findings** | **6** | Describe significant physical examination (PE) and important clinical findings | Lines 89–91 — Physical examinations performed as part of routine clinical care at each visit; findings not systematically recorded as a study outcome; no clinically significant changes identified |
| **Timeline** | **7** | Historical and current information from this episode of care organized as a timeline | Lines 131–143 (individual vignettes), Table 2 (Lines 203–213), and Figures 1A–C and Figure 2 — biochemical trajectories at baseline, 12, 24, and 48 weeks |
| **Diagnostic Assessment** | **8a** | Diagnostic testing (such as PE, laboratory testing, imaging, surveys) | Lines 103–111 — Data Collection and Outcomes sections; ALT, AST, IgG, ALP, bilirubin, albumin, creatinine, LSM by VCTE |
|  | **8b** | Diagnostic challenges (such as access to testing, financial, or cultural) | Not applicable — all patients had established biopsy-proven AIH diagnosis prior to enrollment; no diagnostic challenges encountered |
|  | **8c** | Diagnosis (including other diagnoses considered) | Lines 89 — Biopsy-proven AIH; simplified diagnostic criteria applied (reference 14) |
|  | **8d** | Prognosis (such as staging in oncology) where applicable | Lines 119–121 — Fibrosis staging by LSM (median 21.6 kPa); MELD scores in Table 1; 5 of 7 patients with cirrhosis |
| **Therapeutic Intervention** | **9a** | Types of therapeutic intervention (such as pharmacologic, surgical, preventive, self-care) | Lines 97–99 — Rituximab 1000 mg IV on day 1 and day 15; concomitant immunosuppression continued |
|  | **9b** | Administration of therapeutic intervention (such as dosage, strength, duration) | Lines 97–99 — Rituximab 1000 mg intravenously on days 1 and 15; 48-week follow-up |
|  | **9c** | Changes in therapeutic intervention (with rationale) | Lines 97–99 and Table 1 — Concomitant immunosuppressive therapies adjusted at treating physician's discretion based on clinical response; changes documented per patient in Table 1 |
| **Follow-up and Outcomes** | **10a** | Clinician and patient-assessed outcomes (if available) | Lines 145–157 — Clinician-assessed biochemical outcomes at 12, 24, and 48 weeks. Patient-reported outcomes were not collected (noted as limitation, line 177) |
|  | **10b** | Important follow-up diagnostic and other test results | Lines 145–157 — ALT, AST, IgG at each timepoint; corticosteroid doses in Figure 2 and lines 151–153; Table 2 summarizes response classification |
|  | **10c** | Intervention adherence and tolerability (How was this assessed?) | Lines 161 and 177 — All 7 patients completed 48-week follow-up; rituximab was well tolerated with no serious adverse events, infections, or hepatic decompensation |
|  | **10d** | Adverse and unanticipated events | Line 65 (abstract) and line 161 — No serious adverse events, infections, or hepatic decompensation observed during 48-week follow-up |
| **Discussion** | **11a** | A scientific discussion of the strengths AND limitations associated with this case report | Lines 177 — Strengths and limitations addressed: prospective design, standardized follow-up, granular trajectory data; limitations include small sample size, single center, no control group, confounding by concomitant immunosuppression, no patient-reported outcomes, 48-week follow-up only |
|  | **11b** | Discussion of the relevant medical literature with references | Lines 163–175 — Comparison to Burak et al. (ref 16), IAIHG cohort (ref 12), ColHai registry (ref 13); mechanistic discussion with refs 6, 17–26 |
|  | **11c** | The scientific rationale for any conclusions (including assessment of possible causes) | Lines 167 and 173 — Mechanistic rationale for limited durability: B-cell repopulation within 6–12 months, CD20-negative plasma cell persistence, autoreactive clone re-emergence |
|  | **11d** | The primary "take-away" lessons in a one paragraph conclusion (without references) | Lines 179 — Conclusion paragraph: early biochemical improvement and corticosteroid-sparing confirmed; durability limited; supports further prospective evaluation of B-cell directed therapies |
| **Patient Perspective** | **12** | The patient should share their perspective in one to two paragraphs on the treatment(s) they received | Line 51 — Individual patient perspectives on treatment were not formally collected as part of this prospective case series |
| **Informed Consent** | **13** | Did the patient give informed consent? Please provide if requested | Lines 93–95 — Yes. All patients provided written informed consent. IRB approval: Indiana University IRB Protocol No. 1903320072 |

*Note: Ethnicity was not systematically collected for patients in this cohort. This represents a limitation noted under Item 5a.*
